# Ethnicity-specific associations between the promoter region G-308A polymorphism (rs1800629) of the *TNF*-α gene and the development of end-stage renal disease: An evidence-based meta-analysis and trial sequential analysis

**DOI:** 10.1590/1678-4685-GMB-2024-0077

**Published:** 2025-02-24

**Authors:** Suthiya Anumas, Amarit Tansawet, Pawin Numthavaj, Pattharawin Pattharanitima, Noel Pabalan, Hamdi Jarjanazi, Rungrawee Mongkolrob, Adis Tasanarong, Phuntila Tharabenjasin

**Affiliations:** 1 Thammasat University, Chulabhorn International College of Medicine, Pathumthani, Thailand.; 2 Navamindradhiraj University, Faculty of Medicine Vajira Hospital, Department of Research and Medical Innovation, Bangkok, Thailand.; 3 Mahidol University, Faculty of Medicine Ramathibodi Hospital, Department of Clinical Epidemiology and Biostatistics, Bangkok, Thailand.; 4 Thammasat University, Faculty of Medicine, Division of Nephrology Unit, Rangsit, Pathumthani, Thailand.; 5 Ontario Ministry of the Environment and Parks, Environmental Monitoring and Reporting Branch, Toronto, Ontario, Canada.

**Keywords:** Tumor necrosis factor-alpha, TNF-α, polymorphism, end-stage renal disease, meta-analysis

## Abstract

Tumor necrosis factor-alpha (TNF-α), is partly attributed to pathogenesis of end-stage renal disease (ESRD). Inconsistency of reported associations between *TNF-*α G-308A polymorphism (rs1800629) and ESRD prompted a meta-analysis to obtain more precise estimates. Eleven case-control studies from 11 articles were included. Pooled odds ratios (OR) and 95% confidence intervals (95% CIs) were estimated to evaluate the association. Subgroup analysis was based on ethnicity (Caucasian and Asian). Multiple comparisons were Bonferroni-corrected. Trial sequential analysis (TSA) was implemented to ascertain the reliability of results. Sensitivity analyses and publication bias tests were performed on significant results. There were no significant association (*p* ^*a*^ >0.05) in the overall and ethnic subgroup. Indians, three significant pool ORs (*p* ^*a*^ < 0.01-0.03) showed increased susceptibility to ESRD in homozygous (OR, 6.57; 95% CI, 1.45 to 29.75; *p* ^*a*^ = 0.01), recessive (OR, 6.75; 95% CI, 1.44 to 31.56; *p* ^*a*^ = 0.02), and codominant (OR, 2.06; 95% CI, 1.08 to 3.94; *p* ^*a*^ = 0.03) models. TSA indicated the robustness of such association in the Indian population. The main outcomes were robust without evidence of publication bias. This study showed associations between *TNF-*α G-308A and ESRD are confined to Indians, which are susceptible to ESRD up to approximately 7 times.

## Introduction

End-stage renal disease (ESRD) signifies a chronic and irreversible decline in kidney function, representing the final stage of chronic kidney disease (CKD). Patients with ESRD may require renal replacement therapy, including dialysis (hemodialysis or peritoneal dialysis) or kidney transplantation, in order to maintain homeostasis of body water, electrolytes, and solutes in the extracellular fluid. Without these interventions, ESRD can lead to death due to its associated with various complications. (Modi and Jha, 2006; Abbasi et al., 2010; Kanda et al., 2017; Harrison et al., 2023). In adults, several chronic diseases such as diabetes mellitus type 2, hypertension, and glomerulonephritis, are widely recognized as major risk factors for ESRD worldwide (Evans et al., 2022; GBD Chronic Kidney Disease Collaboration, 2020). These factors could be potentially modifiable targets for attenuating CKD advancement (Hannan et al., 2021). Additionally, genetic factors that are considered unmodifiable have been implicated in CKD progression (Hannan *et al*., 2021). Thus, the identification of genes associated with CKD/ESRD would raise awareness and enable early intervention or the development of targeted drugs to attenuate CKD progression.

Regardless of the etiology, the formation of internal scar tissue or fibrosis in the kidney is a final common histologic finding in most CKD cases, which is the result of a complex inflammatory process (Lee and Kalluri, 2010). Previous studies reported that several inflammatory cytokines, chemokines, and growth factors, including transforming growth factor-β, tumor necrosis factor-alpha (TNF-α), and interleukin (IL)-1 and -6, are involved in this process (Akalin and O’Connell 2010; Taguchi et al., 2021). The expression or the level of cytokine-related inflammatory responses is thought to be governed by genetic variations (Rao et al., 2007). 

In the Chronic Renal Insufficiency Cohort study, biomarkers of inflammation, such as IL-1β, IL-6, TNF-α, C-reactive protein, and fibrinogen, were inversely associated with measures of kidney function (Gupta et al., 2012; Graterol *et al*., 2022). Increased levels of these pro-inflammatory cytokines are strongly associated with a decline in kidney function. Specifically, elevated plasma levels of proinflammatory cytokines, namely TNF-α, pleiotropic cytokines secreted by macrophages/mono-cytes, natural killer cells, mastocytes, adipocytes, and some neoplastic cells, have been found in patients with ESRD and correlate with their mortality (Girndt et al., 1998; Ficek et al., 2006; Wen et al., 2019). In addition, the inflammation pathway driven by TNF-α through TNF receptors 1 and 2 (TNFR1) and 2 (TNFR2) is involved in the pathogenesis and progression of renal injury in CKD (Lousa et al., 2022). 

*TNF-α* gene expression is controlled by the presence of some polymorphisms in its 5-flanking region (promoter region), thereby affecting cytokine levels and thus severity of inflammation (Wilson et al., 1993; Hajeer and Hutchinson 2000). The gene encoding TNF-α is located within the major histocompatibility complex region of chromosome 6p21.3 (Hajeer and Hutchinson 2000). Single nucleotide polymorphism (SNP) in the *TNF-α* gene at position 308 inside the promoter region was proposed to be important functional polymorphism associated with the prevalence of inflammation and its associated phenotypes in ESRD (Hajeer and Hutchinson 2000). The 308A allele has higher transcriptional activity in comparison with the G allele. In the common allele *TNF-1* (G), guanine is present, as opposed to adenine in the relatively rare allele *TNF-2* (A). The *TNF*-2 allele is associated with the ‘high-producer’ TNF-α phenotype (Kroeger et al., 1997; Lee et al., 2000) and is associated with an approximately 6- to 7-fold increase in TNF-α transcription, which may predispose individuals to inflammatory diseases (Wilson *et al*., 1993; Kroeger *et al*., 1997; Lee *et al*., 2000). 

The current literature on the association between the *TNF-α* G-308A polymorphism and the risk of ESRD remains complex and inconclusive. Conflicting findings across studies (Hajeer and Hutchinson, 2000; Jaber et al., 2004; Vázquez-Huerta et al., 2014) underscore the need for a more comprehensive and methodologically rigorous approach to clarify this association. A well-executed meta-analysis can address the limitations of individual studies by providing more precise estimates and potentially identify the associations, which may have been obscured by small sample sizes or methodological variability from a single study. Therefore, we conducted this meta-analysis to investigate the association between the *TNF-α* G-308A (rs1800629) alleles and ESRD risk. 

## Material and Methods

### Selection of studies

To include all relevant primary studies, we performed a MEDLINE search using PubMed, ScienceDirect, Google Scholar, and Mednar for association studies as of January 28, 2024. The search terms applied were “tumor necrosis factor-alpha”, “*TNF-α*”, “cytokine”, “polymorphisms”, “ESRD”, and “end-stage renal disease” as medical subject heading and text, with no language restrictions (Table S1). The references cited in the retrieved articles were also manually screened to identify additional eligible studies. The inclusion criteria were as follows: (i) case-control design that examined the associations between *TNF-α* G-308A and ESRD risk and (ii) provision of sufficient genotype frequency data to enable calculation of odds ratios (ORs) and 95% confidence intervals (CIs). The exclusion criteria were (i) studies without controls or studies whose genotype or allele frequencies were unusable or absent; (ii) those that did not cover the polymorphism or disease in question; and (iii) review articles.

### Data extraction, Hardy-Weinberg Equilibrium (HWE) deviation and study quality assesment

Two investigators (SA and PP) independently extracted data. Disagreements were adjudicated by a third and fourth investigator (RM and PN), who arrived at a consensus. The following key information was obtained from each publication: the first author’s last name, year of publication, country of origin, ethnicity, age and sex ratio in cases and controls, and the quality of the articles (Table 1). The quantitative features of each article were extracted. These included the number of cases and controls, genotype frequencies (*GG*, *GA* and *AA*), minor allele frequency, and HWE. Using the application available at https://ihg.gsf.de/cgi-bin/hw/hwa1.pl., the HWE was assessed, and the *p*-value of the controls from Pearson’s goodness-of-fit χ^2^-square test was reported (Table 2). *P*-value for HWE was set >0.05 indicated HWE-compliance.

**Table 1-:** Characteristics of the included studies in the *TNF-α* G-308A polymorphism associations with ESRD.

	First author	Year	Country	Ethnicity	Age (year)		Sex ratio (Male:Female)		Clark-Baudoin
					Case	Control	Case	Control	
1	Babel	2006	USA	Caucasian	35 ± 20.5	41 ± 8.40	1.70:1	0.75:1	7
2	Bloudickova	2011	Czechoslovakia	Caucasian	65.4 ± 13.1	NA	NA	NA	6
3	Buraczynska	2003	Poland	Caucasian	53.90 (22-78)	46.64 (21-61)	1.16:1	1.50:1	6
4	Buraczynska	2007	Poland	Caucasian	51.04	49.20 ± 9.80	1.31:1	1.12:1	9
5	Guarmeri	2022	Italy	Caucasian	69.01 ± 13.02	55.97 ± 10.99	1.22:1	1.27:1	8
6	Manchada	2006	India	Asian	38.20 ± 13.53	34.96 ± 11.30	4.50:1	1.30:1	7
7	Prakash	2015	India	Asian	NA	NA	4.36:1	4:01	8
8	Ranganath	2009	India	Asian	34.20 ± 10.7	35.80 ± 11.10	7.06:1	5.77:1	9
9	Sharma	2013	India	Asian	39.30 ± 12.6	NA	3.55:1	3.04:1	9
10	Shu	2005	Taiwan	Asian	37.90 ± 11.00	NA	1.45:1	NA	8
11	Singh	2015	India	Asian	51.20 ± 12.9	41.90 ± 14.47	2.70:1	1.40:1	8

*TNF-α*: tumor necrosis factor-alpha; ESRD: end-stage renal disease; *G*: guanine; *A*: alanine; USA: United State of America; NA: not applicable. Age is presented with mean ± standard deviation or median (range).

**Table 2-:** Quantitative features of the included studies of the *TNF-α* G-308A polymorphism associations with ESRD.

	First author	Ethnicity	Case n	Control n	Total n	Power† (%)	Case n			Control n			Minor allele frequency	p-value of HWE
			2,805	3,045	5,850		*GG*	*GA*	*AA*	*GG*	*GA*	*AA*		
1	Babel	Caucasian	103	118	221	31.5	71	27	5	76	33	4	0.181	0.86
2	Bloudickova	Caucasian	483	490	973	**87.6^†^**	360	120	3	330	143	17	0.18	0.76
3	Buraczynska 2003	Caucasian	95	115	210	30.0	62	28	5	86	24	5	0.147	0.066
4	Buraczynska 2007	Caucasian	603	325	928	**82.7^†^**	406	176	21	263	56	6	0.104	0.15
5	Guarmeri	Caucasian	93	213	306	35.9	85	6	2	178	32	3	0.089	0.27
6	Manchada	Asian	231	180	411	51.9	31	103	97	49	126	5	0.377	**5.73 × 10^-11^**
8	Prakash	Asian	300	570	870	**80.0^†^**	233	63	4	392	162	16	0.17	0.88
9	Ranganath	Asian	258	569	827	75.9	204	44	10	498	68	3	0.065	0.68
10	Sharma	Asian	257	200	457	56.2	160	61	36	162	28	10	0.12	**1.86 × 10^-6^**
11	Shu	Asian	297	145	442	50.4	256	39	2	117	24	4	0.168	0.06
11	Singh	Asian	85	120	205	28.0	15	21	49	32	85	3	0.379	**3.25 × 10^-8^**

*TNF-α*: tumor necrosis factor-alpha; ESRD: end-stage renal disease; *n:* number of studies; HWE: Hardy-Weinberg Equilibrium;

*GG*: homozygous *wild-type* guanine-guanine; *GA*: heterozygous *wild type-variant* guanine-alanine; *AA*: homozygous *variant* alanine-alanine;

† high power of data was considered at ≥ 80% (α = 0.05, OR 1.5). Values in bold indicate statistical studies (deviation from HWE).

The Clark-Baudouin scale was used to assess the methodological quality of the included studies (Clark and Baudouin 2006). The Clark-Baudouin criteria include *p*-values, statistical power, correction for multiplicity, sample sizes in cases and controls, genotyping methods, and HWE. Based on this scale, scores <5, 5-6, and ≥7 were considered low, moderate, and high, respectively.

### Data distribution and statistical power

The normality of the data distribution was evaluated by the Shapiro-Wilks test using SPSS (version 20.0; IBM Corp., Armonk, NY, USA). Descriptive statistics and inferential expressions of mean ± standard deviation as well as parametric tests were applied to data showing normal distributions (Gaussian distribution at *p* >0.05). Otherwise, non-parametric tests and medians with interquartile ranges were used. Using the G*Power program (Faul et al., 2007), we evaluated aggregate statistical power, as its adequacy bolsters the level of associative evidence. Statistical power was assessed by assuming an OR of 1.5 with a genotypic risk level of α = 0.05 (two-sided) where high power was considered at ≥80%. 

### Meta-analysis and trial sequential analysis (TSA)

Given the hypothesis of association between *TNF-α* G-308A and risk for ESRD, we estimated the pooled ORs with 95% CIs using genotype frequencies for each study by comparing cases with controls. Z test was used to analyze the statistical significance of OR threshold of *p* ≤ 0.05 (two-tailed). Pooled ORs with 95% CIs were calculated for the following genetic models: (a) homozygous: *AA* and *GG* genotypes compared with *GG*; (b) recessive: *AA* vs. *GA* + *GG*); (c) dominant: *AA* + *GA* vs. *GG*; and (d) codominant: *A* versus *G*. Multiple associative comparisons were further Bonferroni corrected. Subgroup analysis was performed with respect to ethnicity, including Caucasian and Asian populations. Additionally, stratification of specific subgroups was analyzed in the Indian population.

Of note, homozygous refers to having inherited two identical alleles of a genomic marker from each biological parent. Recessive refers to the relationship between an observed trait and the two inherited versions of a gene related to that trait. Dominant assumes one or more copies of the dominant allele increases risk compared to the other allele. Codomiannt refers to a type of inheritance in which two alleles of the same gene are expressed separately to yield different traits in an individual (Horita and Kaneko, 2015; Zhao et al., 2016).

The meta-analysis model choice depends on the presence or absence of heterogeneity (Borenstein et al., 2010). The fixed effect model assumes all the studies in the meta-analysis to have one true effect size, and the observed variations between studies are caused by chance (Egger and Smith, 1997). The random effect model assumes that different studies exhibit substantial diversity and the true effect size may vary from study to study (DerSimonian and Kacker, 2007). Heterogeneity was assessed using the chi-squared-based Q test, the significance threshold of which was set at *p*-value ≤0.10 (Higgins, 2008). Heterogeneity was also quantified with the *I* ^*2*^ statistic that measures variability between studies (Higgins and Thompson, 2002). *I* ^*2*^ values of >50% have more variability than those ≤50%, with 0% indicating zero heterogeneity or homogeneity. Evidence of functional similarities in population features of the studies warranted the use of fixed-effects model (Mantel and Haenszel, 1959); otherwise, the random-effects model (DerSimonian and Laird, 1986) was used .

Sensitivity analysis, which involved omitting one study at a time and recalculating the pooled OR, was used to test the robustness of the summary effects. Tests for publication bias were used to evaluate the influence of small-study specific effects (Ioannidis, 2008) and were applied only to comparisons that showed associative significance (*p* <0.05) and passed Bonferroni correction. We selected a publication bias test based on the data distribution. In the presence of normally distributed data, we used the Egger’s test (Egger et al., 1997); otherwise, we used the Begg-Mazumdar test (Begg and Mazumdar 1994). Publication bias was examined visually by the funnel plot and was considered significant at *p*-value <0.05. 

Data were analyzed using Review Manager 5.3 (Cochrane Collaboration, Oxford, England), SIGMASTAT 2.03, SIGMAPLOT 11.0 (Systat Software, San Jose, California, United States), and WINPEPI (Abramson, 2004).

TSA was carried out to validate whether the results of the meta-analysis could reach definite conclusions by using the TSA software version 0.9.5.10 (Copenhagen Denmark: The Copenhagen Trial Unit, Center for Clinical Intervention Research, 2021). In this study, we performed TSA on the genetic models and the settings of parameters were listed as follows. Relative risk reduction was set to low risk of bias based. The type 1 error and power were set as 0.05 and 0.80, respectively. Regarding sample size estimation, a model variance-based preset value was selected for heterogeneity correction. Two-sided 5%-symmetrical O’Brien- Fleming significance and futility boundaries were constructed. When the cumulative Z-curve crossed the TSA monitoring boundary or exceeded the required information size line, it confirms the presence of robust evidence. Otherwise, additional trials were considered to be necessary.

## Results

### Characteristics of the included studies 

**Figure 1-:**
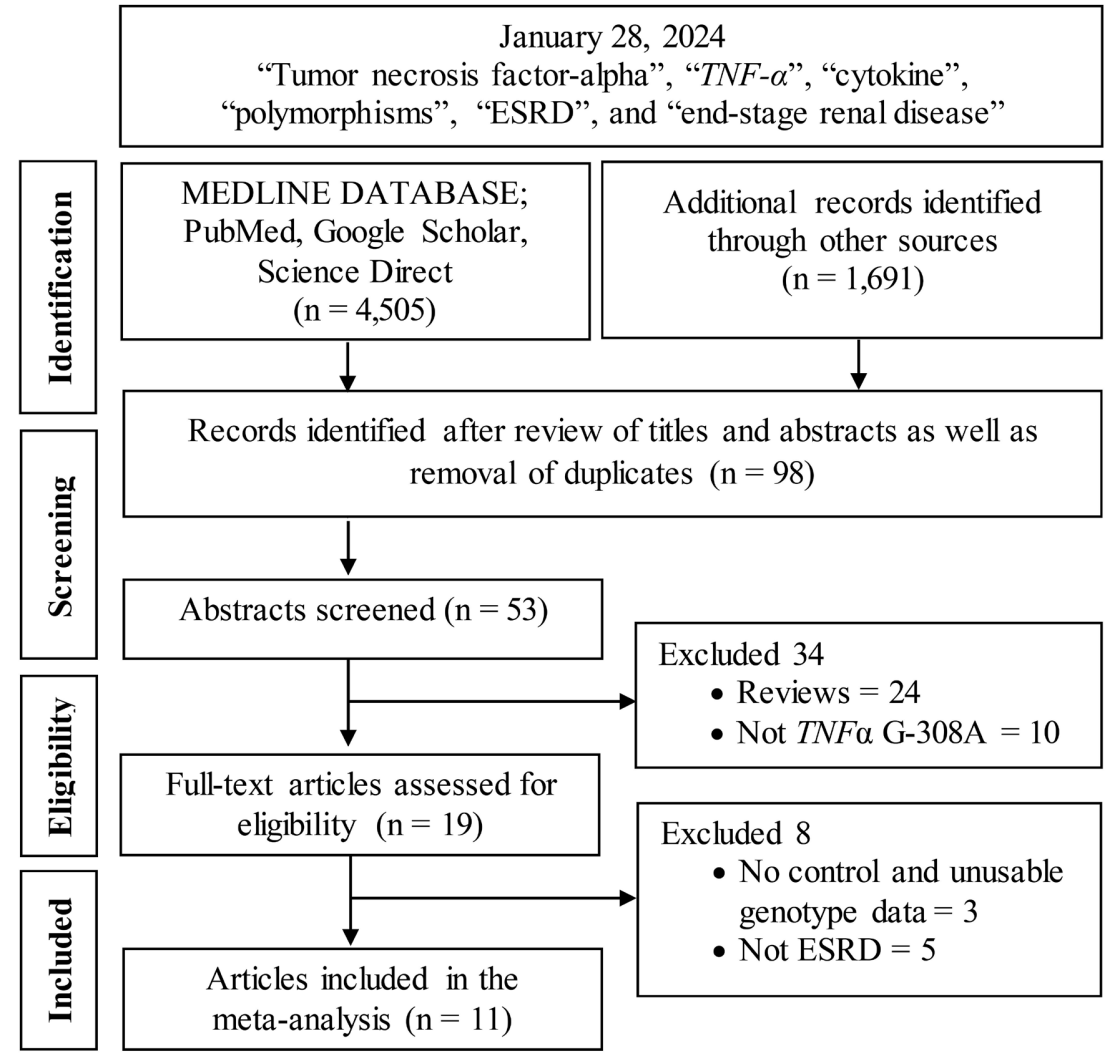
Flowchart on the selection of studies for inclusion in the meta-analysis.

According to the Preferred Reporting Items for Systematic Reviews and Meta-Analyses (PRISMA) guidelines (Moher et al., 2009) (Table S2), Figure 1 summarizes the study selection process in a PRISMA sanctioned flowchart. The initial search yielded 6,196 citations, which were reduced to 98 after title and abstract reviews and removal of duplicates. The full texts of 19 articles were obtained, of which eight were excluded because they did not conform to the inclusion criteria. A manual search of the reference lists yielded no additional articles. Table 1 lists the 11 articles with 11 studies included in this meta-analysis (Buraczynska et al., 2003; Shu et al., 2005; Babel et al., 2006; Manchanda et al., 2006; Buraczynska *et al*., 2007; Ranganath et al., 2009; Bloudíčková et al., 2011; Sharma et al., 2013; Singh et al., 2015; Prakash et al., 2016; Guarneri et al., 2022). From the 11 studies, the subjects in five studies (Buraczynska *et al*., 2003; Babel *et al*., 2006; Buraczynska *et al*., 2007; Bloudíčková *et al*., 2011; Guarneri *et al*., 2022) included a Caucasian population (1,462 cases/1,381 controls) and the remaining six studies were on Asian populations (1,428 cases/1,784 controls). Among Asian studies, five of six focused on the Indian population (Manchanda *et al*., 2006; Ranganath *et al*., 2009; Sharma *et al*., 2013; Singh *et al*., 2015; Prakash *et al*., 2016). The age of the subjects in the case and control groups ranged from 34 to 69 years and 34 to 56 years, respectively. The mean and standard deviation values of the normally distributed Clark-Baudouin scores (Shapiro-Wilks: *p* = 0.097) were 7.23 and 1.10, respectively, with most (90.9%) of the articles scoring >7. These values indicated the high methodological quality of the included studies.

Table 2 presents the quantitative features of the studies included. The total sample sizes in the case and control were 2,805 and 3,045, respectively. The three articles (Buraczynska et al., 2007; Bloudíčková et al., 2011; Prakash et al., 2016) had high statistical powered (80.0-87.6%), including 1,386 cases and 1,385 controls. Controls in the three studies deviated from HWE (Manchanda et al., 2006; Sharma et al., 2013; Singh et al., 2015).

### Meta-analysis and TSA outcomes

**Figure 2-:**
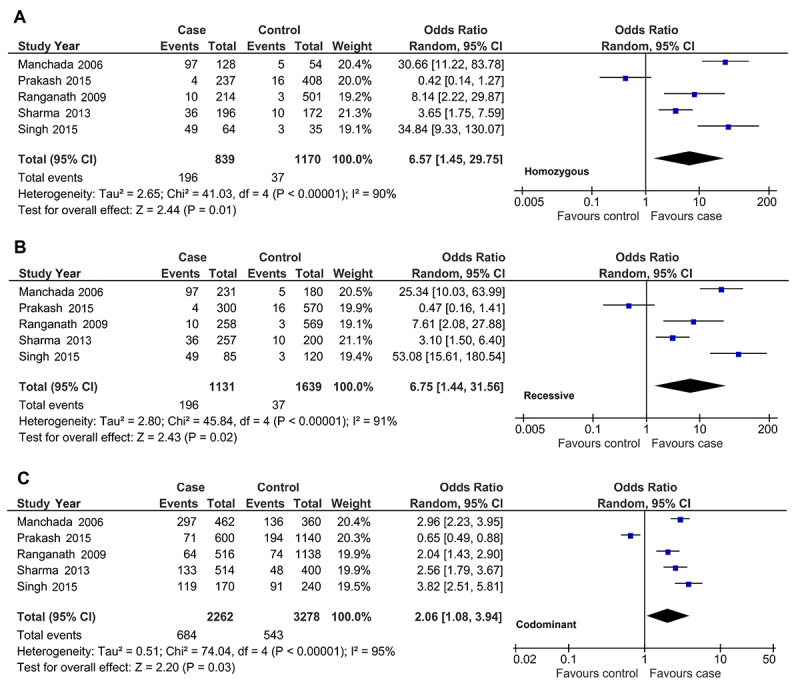
The forest plot in the three significant outcomes of associations of *TNF-α* 308 *G/A* gene polymorphism and ESRD in Indian population-specific subgroup. A) homozygous, B) recessive, and C) codominant model. *TNF-α*, *tumor necrosis factor-alpha gene*; ESRD, end-stage renal disease; G, guanine base in DNA; A, alanine base in DNA; CI, confidence interval; df, degree of freedom; *I*_*2*_, measure of variability.

There were no significant associations of *TNF-α* with ESRD in overall (ORs, 1.19-2.14; 95% CI, 0.75 to 5.96; *I* ^*2*^, 87-94%; *p* ^*a*^ = 0.14-0.35), Caucasian (ORs, 0.92-1.04; 95% CI, 0.37 to 2.69; *I* ^*2*^, 61-88%; *p* ^*a*^ = 0.86-0.99), and Asian (ORs, 1.41-4.09; 95% CI, 0.80 to 18.78; *I* ^*2*^, 89-95%; *p* ^*a*^ = 0.07-0.22) subgroup. Since only one Taiwanese (Shu et al., 2005) article contributed to Asians, we thus meta-analyzed by stratification of Indian s (*n* = 5). By specification of Indian population, three significant association ORs with passed Bonferroni correction were observed in the homozygous (OR, 6.75; 95% CI, 1.45 to 29.75; *I* ^*2*^, 90%; *p* ^*a*^ = 0.01), recessive (OR, 6.75; 95% CI, 1.44 to 31.56; *I* ^*2*^, 91%; *p* ^*a*^ = 0.02), and codominant (OR, 2.06; 95% CI, 1.08 to 3.94; *I* ^*2*^, 95%; *p* ^*a*^ = 0.03) comparisons, all of which were derived from random-effects and indicated an elevated risk of ESRD (Table 3, Figure 2).

**Table 3-:** Summary effects of *TNF-α* G-308A polymorphism with ESRD.

	*n*	Test of association			Test of heterogeneity	
		OR	95% CI	*p* ^*a*^	*p* ^*het*^	*I* ^*2*^ (%)
**Overall**						
Homozygous	11	2.14	0.78-5.87	0.14	< 0.00001	88
Recessive	11	2.11	0.75-5.96	0.16	< 0.00001	89
Dominant	11	1.19	0.82-1.74	0.35	< 0.00001	87
Codominant	11	1.30	0.84-2.02	0.23	< 0.00001	94
**Caucasian**						
Homozygous	5	0.99	0.37-2.69	0.99	0.02	67
Recessive	5	0.92	0.37-2.29	0.86	0.04	61
Dominant	5	0.97	0.55-1.72	0.92	0.00001	88
Codominant	5	1.04	0.63-1.71	0.89	0.00001	87
**Asian**						
Homozygous	6	3.97	0.88-17.83	0.07	< 0.00001	91
Recessive	6	4.09	0.89-18.78	0.07	< 0.00001	91
Dominant	6	1.41	0.82-2.45	0.22	< 0.00001	89
Codominant	6	1.58	0.80-3.11	0.19	< 0.00001	95
**Indian**						
Homozygous	5	**6.57**	**1.45-29.75**	**0.01^†^**	< 0.00001	90
Recessive	5	**6.75**	**1.44-31.56**	**0.02^†^**	< 0.00001	91
Dominant	5	1.64	0.89-3.00	0.11	< 0.00001	89
Codominant	5	**2.06**	**1.08-3.94**	**0.03^†^**	< 0.00001	95

*TNF-α*: tumor necrosis factor-alpha; ESRD: end-stage renal disease; *G*: guanine; *A*: alanine; *n*: number of studies; OR: odds ratio; CI: confidence interval; *p* ^*a*^: *p*-value for test of association; *p* ^*het*^: *p*-value for heterogeneity; *I* ^*2*^: measure of variability expressed in %, **†** significant core outcomes passed Bonferroni correction; All comparisons underwent the random effects anaylsis.

**Figure 3-:**
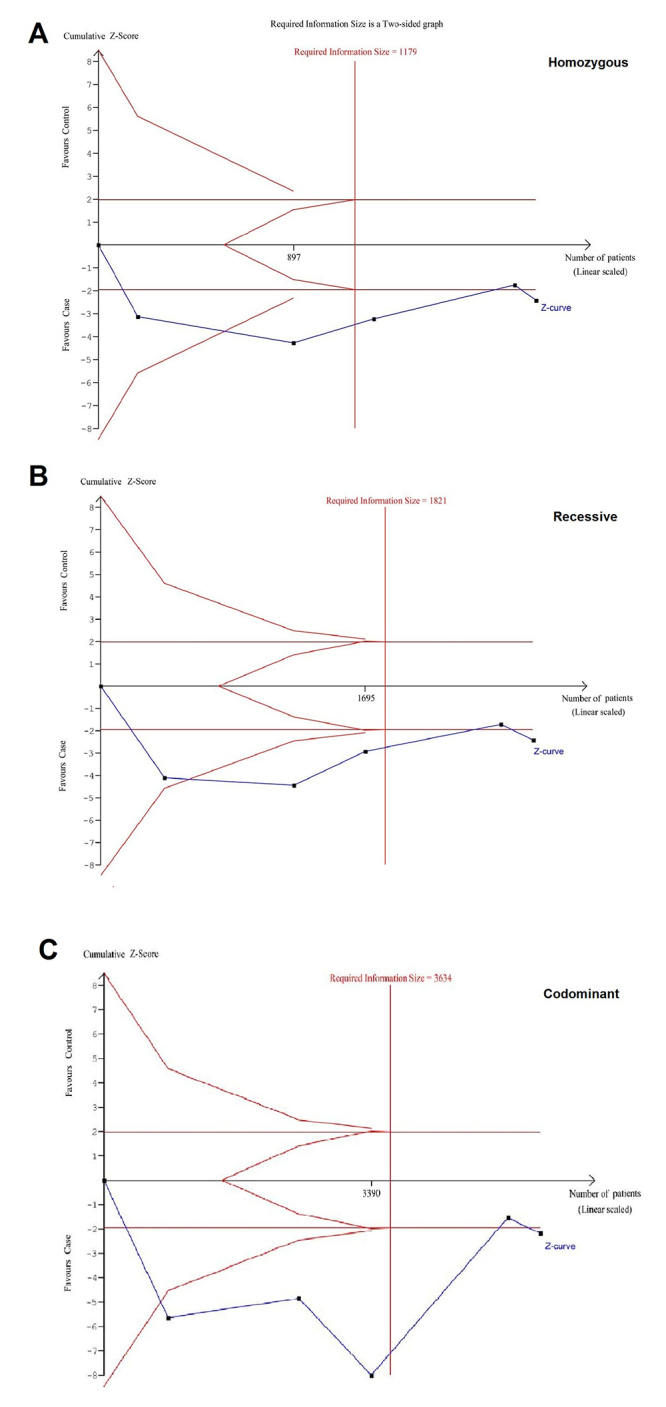
Trial sequential analysis of core findings in Indians based on genetic models of homozygous (A), recessive (B), and codominant (C).

As for TSA estimation, it was conducted on analysis of Indian population (homozygous, recessive, and codominant). As shown in Figure 3, the cumulative Z-curves in all three genetic models crossed the significance boundary and the required information size was reached, suggesting adequate sample size. TSA results support association between *TNF-α* G-308A polymorphism and the development of ESRD. No further relevant trials were needed. Therefore, definite results of meta-analysis can be obtained for Indians.

**Figure 4 -:**
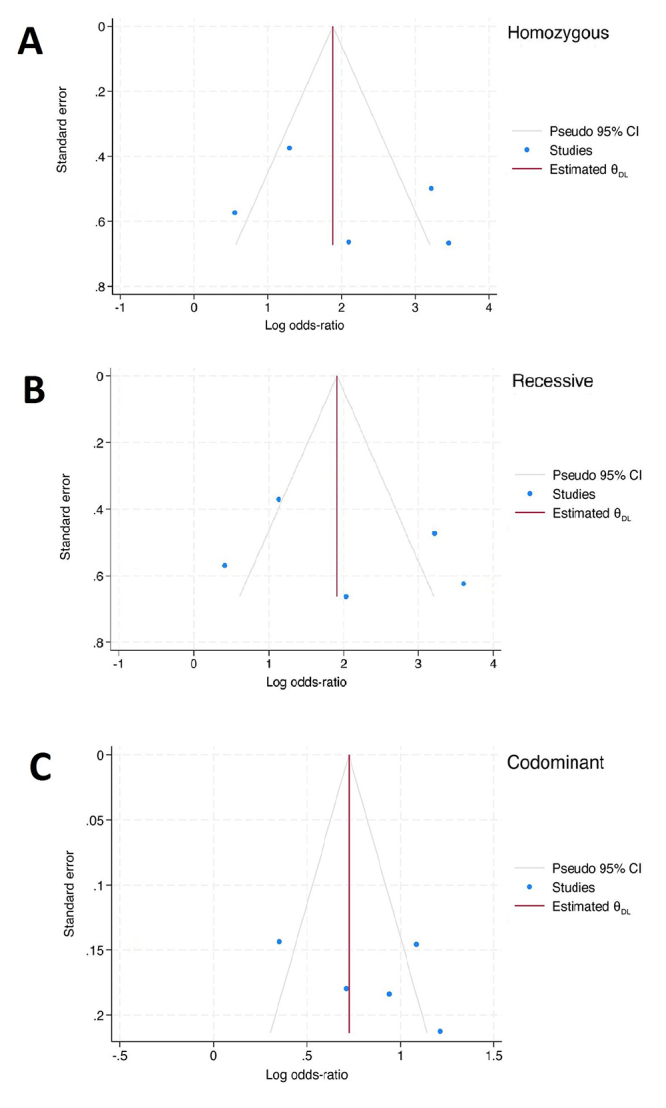
Begg-Mazumdar funnel plot with pseudo 95% confidence limits for publication bias test under homozygous (A), recessive (B), and codominant (C) model in Indian ethnicity population. CI, confidence interval.

Table 4 presents the results of the publication bias assessment and sensitivity analysis for the three significant outcomes, all of which showed no significant publication bias in the *TNF-α* rs1800629 variant (Begg-Mazumdar test: *p* = 0.62-0.63). The shape of the Begger’s funnel plot seems to be symmetric by visual inspection and did not show any evidence of publication bias under the three genetic models (Figure 4). Robustness of sensitivity was found only in the homozygous and recessive genetic models.

**Table 4 -:** Assessment of sensitivity and publication bias test of core outcome.

Comparison (n)	Genetic model	Sensitivity outcome	Shapiro-Wilks test *p*-value	Normal distribution	Publication bias test	***p*-value**	Evidence of publication bias
Indian (5)	Homozygous	Robust	< 0.001	No	Begg-Mazumdar	0.624	No
	Recessive	Robust	< 0.001	No	Begg-Mazumdar	0.620	No
	Codominant	Non-Robust	< 0.001	No	Begg-Mazumdar	0.633	No

*n*: number of studies.

## Discussion

In this study, the association between *TNF-*α rs1800629 and ESRD risk depended on usable genotype data from the literature, which then used an arsenal of meta-analytical tools, including subgrouping, publication bias, and sensitivity analysis. Furthermore, Bonferroni correction can minimize the risk of false positives (Armstrong, 2014) and obtain more precise data synthesis. 

To our knowledge, this is the first meta-analysis to address the association between *TNF-α* polymorphism and ESRD risk. Our results indicated *TNF-α* G-308A polymorphism (rs1800629) was significantly associated with an increased risk of ESRD only in Indian-subspecific Asian populations but not in the overall and Caucasian populations. Based on the significant pooled OR obtained, individual indian with ESRD that exhibit polymorphism in the homozygous, recessive, and codominant models are more likely to develop ESRD than those without the SNP.

The ESRD has been recognized to be involved in chronic inflammation process, which is partly mediated by inflammatory cytokine, TNF-α (Akchurin and Kaskel, 2015). Regarding the genotypes and alleles of the *TNF α -308G >A* polymorphism, the highest to the lowest TNF-α cytokine production or phenotype (Hutchinson et al., 1998) correspond to *AA*, *GA*, and *GG* genotypes, respectively. Moreover, the level of albuminuria as an ESRD indicator with the *AA* genotype was also observed to have higher levels of circulating TNF-α than those with the *GG* genotype (Umapathy et al., 2018). Altogether, it could be postulated that the TNF-α level of individuals with the mutant genotype is higher than that of individuals with the wild-type genotype, which is capable of contributing to ESRD development.

Our study showed a significant association between *TNF-α* SNP in homozygous, recessive, and codominant models and ESRD, which seems to be supported by the TNF-α genotype phenotype concept. Interestingly, we found that the influence of *TNF-α* polymorphisms on ESRD risk depended on ethnic differences, which are consistent with a previous meta-analysis of eight studies by Chen et al. (2020). Ethnicity-stratified analysis revealed no association between *TNF-α* rs1800629 variant and risk of acute kidney injury in Caucasians. This finding implies that the distribution of alleles could partially explain the susceptibility differences in the ethnic background. 

Besides genetic variations, patients with ESRD usually have a certain number of comorbid factors such as malnutrition status, cardiovascular diseases, hypertension, and diabetes. These factors influence on immune dysregulation and inflammatory process activation in ESRD (Akchurin and Kaskel, 2015; Eiam-Ong and Sitprija, 2002). With the complexity of comorbid diseases and ESRD, it is well-documented that diabetic nephropathy, or in combination with hypertensive nephropathy, is the most common cause of ESRD (Ghaderian et al., 2015). TNF-α is regarded as an important factor in obesity-associated insulin resistance (Li et al., 2003; Liu et al., 2020). In a cross-sectional study, Alzamil (2020) demonstrated that elevated serum TNF-α levels were strongly related to obesity and HbA1c levels in patients with type 2 diabetes mellitus. At the cellular level, elevation of TNF-α levels produced by adipocytes and/or peripheral tissues induces insulin resistance by impairing the insulin signaling pathway through serine phosphorylation (Akash et al., 2018). 

As for the association of diabetic nephropathy (DN) as a major risk factor for ESRD and *TNF-α* SNP, a previous meta-analysis showed *TNF-α-308G/A* polymorphism was significantly related to an increased risk of DN in homozygous and recessive models, and similar results were obtained only in Asian groups (Liu et al., 2020). In addition, a previous report (Statista, 2022 by John Elflein) revealed that first and second country with the highest number of diabetics worldwide are China and India, respectively. In the present study, a significant association between *TNF-α-308G/A* polymorphism and risk of ESRD was found only in the Indian population, which may be partially explained by comorbid disease factors. Moreover, the differences between ethinicity could be interpreted partially by the influence of gene-environment factors (e.g. diet, life style, exposure to toxin) interaction in process of ESRD. Also, significant association effects observed in Indian population may be due to a variation of linkage disequillibrium (LD) pattern between populations. Specifically, *TNF-α* G308A polymorphism in Indians might be in LD with other genetic variants that have more influence on the risk of ESRD than the other populations, leading to population-specific associations. For example, Morar et al. (2007) reported information on the LD pattern of markers in the *TNF-α* G308A region with susceptibility to schizophrenia within 30 kb high LD region/block or elsewhere in histocompatibility complex. In addition, the influence of the *TNF-α* G308A might be unmasked by the presence of other as-yet unidentified reasons involved in ESRD development. Thus far, the possible effect of *TNF-α* gene promoter polymorphism on the ESRD and clinical outcome remains to be elucidated.

Genetics plays a significant role in the pathogenesis of various diseases, including cancer, glomerulonephritis, and ESRD. Despite the availability of medications with evidence of slowing CKD progression, the incidence of ESRD remains high. Based on our findings, we identified genes associated with ESRD that could potentially guide the development of targeted drugs that can attenuate CKD progression and open new avenues for therapeutic interventions in the future.

Pointing out the strengths and limitations contextualizes the interpretation of the results of our meta-analysis. The important strengths include: (i) 9 of the 11 included articles (82%) had high Clark-Baudouin scores (≥ 5), indicating high quality of the component studies; (ii) a large sample size in both case and control allows a more precise and accurate interpretation; (iii) consistent results were found in the Indian subgroup, indicating an increased risk of ESRD; (iv) the main outcomes showed no publication bias and had high stability; and (v) TSA technique used in this study can strengthen meta-analysis outcomes as it controls type-I and type-II errors. Contrasting with these strengths are the following limitations of our study: (i) the majority (8/11:73%) of the included studies were underpowered (< 80%); (ii) the influence of *TNFα* rs1800629 does not preclude effects from other polymorphisms in proximity to *TNF*α given the complete LD between them; (iii) the significant outcomes of India might not be representative of all Asian populations; (iv) high heterogeneity (a random-effects model) for data analysis represented by *I* ^*2*^ was found in all analyses; (v) significant Indian outcomes were lost if HWE was applied; and (vi) the effects of gene-gene and gene-environment interactions were not addressed due to lack of adequate data.

The present meta-analysis suggests that the *TNF-α-308G/A* polymorphism may contribute to the susceptibility of ESRD in homozygous, recessive, and codominant genetic models among Asian subgroups, specifically Indian populations. Concurrently, no significant association was observed among the Caucasians. The interpretation of our findings is based on the lack of publication bias and robustness of the outcome.

However, our results identified only Indians. This would probably require further studies on other populations with larger sample sizes. Moreover, haplotype analyses should be performed to delineate the combined effects and elucidate how genetic variations in several genes cooperate in the etiology of ESRD. Additional well-designed component studies (including meta-analyses) considering the other confounding factors of ESRD would confirm or modify our results and add to the existing knowledge about the association between *TNF-α-308G/A* polymorphism and risk of ESRD.

## Supplementary Material

The following online material is available for this article:

Table S1 **-**
Database search algorithms for *TNF-α* G-308A promoter polymorphisms in ESRD.

Table S2 **-**
Checklist of PRISMA guidelines.
