# Supplementary material for: Ethnicity-specific associations between the promoter region G-308A polymorphism (rs1800629) of the TNF-α gene and the development of end-stage renal disease: An evidence-based meta-analysis and trial sequential analysis
Source: Genet Mol Biol. 2025 Feb 24;48(1):e20240077. doi: 10.1590/1678-4685-GMB-2024-0077 (PMC11912548; doi:10.1590/1678-4685-GMB-2024-0077)
Supplement: Table S1 - [file 1415-4757-GMB-48-01-e20240077-s1.pdf]

*Genetics and Molecular Biology*
https://doi.org/10.1590/1678-4685-GMB-2024-0077

# Suplementary Material to "Ethnicity-specific associations between the promoter region G-308A polymorphism (rs1800629) of the *TNF-α* gene and the development of end-stage renal disease: an evidence-based meta-analysis and trial sequential analysis"

**Table S1** - Database search algorithms for *TNF-α* G-308A promoter polymorphisms in ESRD.

| **Database address** | **Search strings** | | | |
|---|---|---|---|---|
| Search as of 28 January 2024 | **1** | **2** | **3** | **4** |
| MEDLINE using PubMed | 40 | 46 | 35 | 41 |
| https://www.ncbi.nlm.nih.gov/pubmed/ | | | | |
| Science Direct [research article] | 216 | 1,940 | 209 | 1,975 |
| https://www.sciencedirect.com/search | | | | |
| Google Scholar [all in title] | 1 | 1 | 0 | 1 |
| https://scholar.google.ca/ | | | | |
| **Grey literature** | | | | |
| Mednar (deep web) | 220 | 503 | 394 | 574 |
| http://mednar.com/mednar/desktop/en/search.html | | | | |

**String search terms**

1. *TNF-α* polymorphisms cytokine ESRD
2. *TNF-α* polymorphisms cytokine end-stage renal disease
3. Tumor necrosis factor-alpha cytokine polymorphisms ESRD
4. Tumor necrosis factor-alpha cytokine polymorphisms end-stage renal disease
